# Supplementary material for: Targeting Cholesterol-Dependent Piezo1 Activation Impairs Amoeboid Migration in Melanoma Cells
Source: bioRxiv. 2025 Jul 17:2025.07.11.664494. Preprint. [Version 1] doi: 10.1101/2025.07.11.664494 (PMC12338549; doi:10.1101/2025.07.11.664494)
Supplement: Supplement 4 [file NIHPP2025.07.11.664494v1-supplement-4.pdf]

bioRxiv preprint doi: https://doi.org/10.1101/2025.07.11.664494; this version posted July 17, 2025. The copyright holder for this preprint (which was not certified by peer review) is the author/funder, who has granted bioRxiv a license to display the preprint in perpetuity. It is made available under aCC-BY 4.0 International license.

VIDEOS

Video S1. A vehicle treated melanoma cell confined to ~3 µm by a PDMS ceiling coated with BSA (1%) adopt a leader mobile (LM) phenotype.

Video S2. A Fluvastatin treated melanoma cell confined to ~3 µm by a PDMS ceiling coated with BSA (1%) adopt a no leader (NL) phenotype.

SOURCE DATA

Source data 1. Categorical data plotted in Figures 1E, 1I, 1K, 5B, 5F, and S1A and associated χ^2 tests.

bioRxiv preprint doi: https://doi.org/10.1101/2025.07.11.664494; this version posted July 17, 2025. The copyright holder for this preprint (which was not certified by peer review) is the author/funder, who has granted bioRxiv a license to display the preprint in perpetuity. It is made available under aCC-BY 4.0 International license.

SUPPLEMENTAL INFORMATION

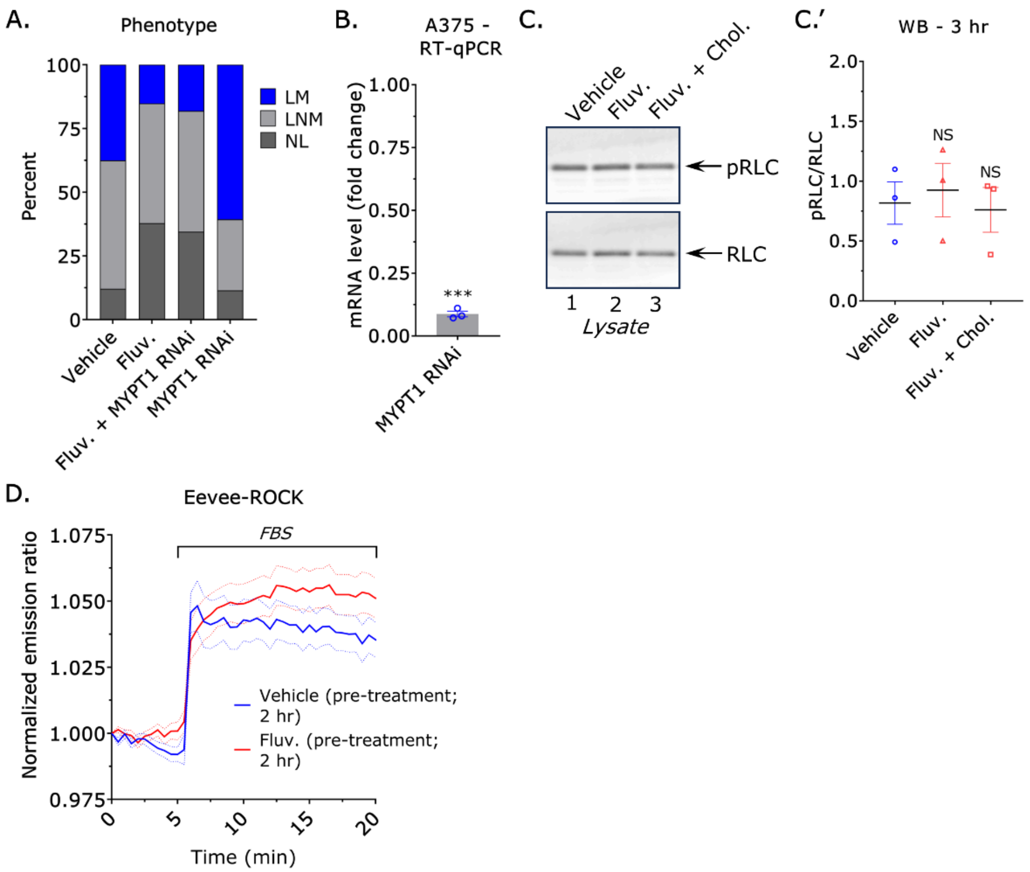

Supplemental Figure 1. Cholesterol depletion does not impair actomyosin contractility. A. Percentage of leader mobile (LM), leader non-mobile (LNM), and no leader (NL) for vehicle (*n* = 334), Fluvastatin (10 µM; *n* = 340) ($\chi^2 < 0.0001$), Fluvastatin (10 µM) + MYPT1 RNAi (*n* = 530) ($\chi^2 < 0.0001$), and MYPT1 RNAi (*n* = 320) ($\chi^2 < 0.0001$) cells. **B.** Fold change in mRNA after MYPT1 RNAi, as measured by RT-qPCR (mean +/- SEM). **C.** Western blots against phosphorylated regulatory light chain (pRLC; S19) and regulatory light chain (RLC) from lysates of vehicle, Fluvastatin (10 µM; 3 hr), and Fluvastatin (10 µM; 3 hr) + cholesterol (1 mM; 3 hr) treated cells (mean +/- SEM). A Dunnett's multiple comparison test was used to determine statistical significance. **D.** Normalized emission ratio for Eevee-ROCK from starved cells pre-treated (2 hr) with vehicle or Fluvastatin (10 µM). Fetal bovine serum (FBS; 10%) was added after 5 min (mean +/- SEM). Significance levels: * - $p \leq 0.05$, ** - $p \leq 0.01$, *** - $p \leq 0.001$, and **** - $p \leq 0.0001$. See also source data 1.
